# Lifespan of restriction-modification systems critically affects avoidance of their recognition sites in host genomes

**DOI:** 10.1186/s12864-015-2288-4

**Published:** 2015-12-21

**Authors:** Ivan Rusinov, Anna Ershova, Anna Karyagina, Sergey Spirin, Andrei Alexeevski

**Affiliations:** Faculty of Bioengineering and Bioinformatics, Lomonosov Moscow State University, Moscow, 119992 Russia; Belozersky Institute of Physico-Chemical Biology, Lomonosov Moscow State University, Moscow, 119992 Russia; Gamaleya Center of Epidemiology and Microbiology, Moscow, 123098 Russia; Institute of Agricultural Biotechnology, the Russian Academy of Sciences, Moscow, 127550 Russia; Scientific Research Institute for System Studies, the Russian Academy of Science (NIISI RAS), Moscow, 117281 Russia

**Keywords:** Restriction-modification systems, Site avoidance, Methylation, Restriction endonuclease, Prokaryotic genome

## Abstract

**Background:**

Avoidance of palindromic recognition sites of Type II restriction-modification (R-M) systems was shown for many R-M systems in dozens of prokaryotic genomes. However the phenomenon has not been investigated systematically for all presently available genomes and annotated R-M systems. We have studied all known recognition sites in thousands of prokaryotic genomes and found factors that influence their avoidance.

**Results:**

Only Type II R-M systems consisting of independently acting endonuclease and methyltransferase (called ‘orthodox’ here) cause avoidance of their sites, both palindromic and asymmetric, in corresponding prokaryotic genomes; the avoidance takes place for ~ 50 % of 1774 studied cases. It is known that prokaryotes can acquire and lose R-M systems. Thus it is possible to talk about the lifespan of an R-M system in a genome. We have shown that the recognition site avoidance correlates with the lifespan of R-M systems. The sites of orthodox R-M systems that are encoded in host genomes for a long time are avoided more often (up to 100 % in certain cohorts) than the sites of recently acquired ones. We also found cases of site avoidance in absence of the corresponding R-M systems in the genome. An analysis of closely related bacteria shows that such avoidance can be a trace of lost R-M systems. Sites of Type I, IIС/G, IIM, III, and IV R-M systems are not avoided in vast majority of cases.

**Conclusions:**

The avoidance of orthodox Type II R-M system recognition sites in prokaryotic genomes is a widespread phenomenon. Presence of an R-M system without an underrepresentation of its site may indicate that the R-M system was acquired recently. At the same time, a significant underrepresentation of a site may be a sign of presence of the corresponding R-M system in this organism or in its ancestors for a long time. The drastic difference between site avoidance for orthodox Type II R-M systems and R-M systems of other types can be explained by a higher rate of specificity changes or a less self-toxicity of the latter.

**Electronic supplementary material:**

The online version of this article (doi:10.1186/s12864-015-2288-4) contains supplementary material, which is available to authorized users.

## Background

Restriction-modification (R-M) systems were discovered and characterized as bacterial systems defending cells from an invasion of foreign DNA, *e.g.*, phage DNA [1, 2]. R-M systems are divided into four types (I–IV) [3]. Classical R-M systems of Types I–III include a DNA methyltransferase (MTase) and a restriction endonuclease (REase) [4]. MTase methylates specific DNA sequences (recognition sites) in the host genome. REase recognizes the same unmethylated site and cleaves the DNA. As a result, the methylated host DNA remains intact whilst any foreign DNA with unmethylated recognition sites is cleaved. In contrast to classical R-M systems, methyl-directed ones of Type IV and IIM include only REases, which cleave modified (*e.g.*, methylated) sites preventing attacks of phages that have acquired modifications of the genomic DNA as an antirestriction strategy [5].

Besides defence from phages, R-M systems may participate in: (i) containment of the horizontal gene transfer [6] and maintenance of bacterial or archaeal population structure [7, 8], (ii) the regulation of gene expression by site methylation [9–11], (iii) modulation of the genome recombination [12].

R-M systems are widely spread among prokaryotes: they have been found or predicted with computational methods in the vast majority of bacterial and archaeal genomes [13]. Besides prokaryotes, R-M systems were found in *Chlorella* viruses [14–16] and a few other eukaryotic viruses [17, 18].

Acquirement and loss of an R-M system is a routine event in the evolution of most bacteria and archaea. A bacterium can occasionally acquire an R-M system with a new specificity by the horizontal gene transfer [19–21] or by the alteration of the DNA recognition domain of an existing R-M system by an intragenomic recombination [22–24] or point mutations [10]. A large number of corrupted R-M system genes in REBASE [25] testify in favor of a rather frequent loss of R-M systems. It allows us to talk about their lifespan in bacteria. R-M system gain and loss is observed even on the level of strains of the same species, strains often carry different sets of R-M systems. Well studied examples are *Neisseria meningitidis* [6], *Streptococcus pneumoniae* [7], *Helicobacter pylori* [26].

R-M system recognition site avoidance is one of numerous antirestriction strategies of phages [4]. The avoidance was revealed in many phage genomes [27, 28]. A similar effect was found in prokaryotic genomes. Karlin and co-workers [29] supposed that the underrepresentation of short palindromes in prokaryotic genomes is associated with a selection aimed at reducing the number of restriction sites. It was shown that some palindromes are the most underrepresented “words” among all possible “words” of certain length in a genome. The known sites of R-M systems were often found among the most underrepresented palindromes [30–33]. The avoidance of R-M system sites was explained by occasional failure of site methylation resulting in DNA cleavage at this site. In the light of recent data on the modulation of gene expression by R-M systems [10], it is possible that unfavorable methylation of particular sites could also provoke their removal.

In this work we study recognition site underrepresentation for thousands of R-M systems in thousands of prokaryotic genomes. All data on cleavage sites of REases were obtained from REBASE [25]. Underrepresentation of the sites was estimated in those genomes that encode corresponding R-M systems. Such site-genome pairs are called ‘actual pairs’. We found two factors, R-M system type and its lifespan, that seem to be the most essential and can basically explain statistical data on site underrepresentation.

## Methods

### R-M system recognition sites

The list of studied R-M systems recognition sites is available in Additional file 1. This list consists of (1) all recognition sites of restriction endonucleases (REases) from REBASE [25] and (2) all 4 bp and 6 bp palindromic sequences that are not in the above list from REBASE (there are two 4 bp and eight 6 bp such palindromes). Each considered site was attributed to one of six groups corresponding to R-M system types: Type I, Type IIC/G, Type IIM, Type II except IIC/G and IIM, Type III, and Type IV. Type II R-M systems except Type IIC/G and IIM ones are called “orthodox” here for shortness. Note that our “orthodox” sites include both palindromic and non-palindromic recognition sites, in contrast to the terminology used by Pingoud *et al.* [34]. Ten added palindromes are considered as sites of orthodox Type II R-M systems because the majority of non-degenerate palindromes of length four and six belong to this type. Statistical data on recognition sites are shown in Table 1.

**Table 1:** Statistical data on recognition sites of R-M systems of different types. Numbers of degenerate sites are shown in parenthesis

Type of R-M system	# of palindromic sites	# of non-palindromic sites	Total # of sites
I	5 (5)	171 (171)	176 (176)
Orthodox II	186 (93)	81 (23)	267 (116)
IIC/G	4 (4)	102 (57)	106 (61)
IIM	2 (2)	8 (7)	10 (9)
III	0	39 (8)	39 (8)
IV	2 (2)	1 (1)	3 (3)
Total	199 (106)	402 (267)	601 (373)

In 29 cases the same nucleotide sequence can be a recognition site of REases of different types. Sites of orthodox Type II REases that are at the same time sites of REases of Type I (1 site), Type IIC/G (14 sites), Type IIM (7 sites), or Type III (5 sites) are counted in Table 1 as orthodox Type II. Two sites of Type IIC/G REases that are also sites of Type III REases are counted as Type IIC/G.

Recognition sites can be non-degenerate (for example, GATC) or degenerate (for example, GANTC or YATR, here N is for any nucleotide, Y is for C or T, and R is for A or G).

### Genomic sequences

In REBASE genome section [25], a list of R-M systems is available for several thousand prokaryotic genomes. In this work, we used only genomes with assembled chromosomes according to NCBI genome assemblies information [35]. The used genome set includes 1980 genomes of Bacteria and 134 genomes of Archaea. These genomes belong to 1213 species of 628 genera. Sequences of the genomes were downloaded from GenBank [36], totally 3882 sequences of chromosomes and plasmids. The list of all sequence IDs is available in Additional file 2; in that list, genomes encoding no R-M systems are marked “No” in the column “Genome with R-M systems”. In this paper “genome” means sequences of all chromosomes and plasmids of a certain organism.

According to REBASE (April 2015) 1859 bacterial and 133 archaeal genomes from our genome set encode at least one R-M system and 121 bacterial genomes and one archaeal genome encode no R-M systems.

Genomes of eukaryotic viruses were downloaded from NCBI [35], the list of all sequence IDs is available in Additional file 3. Three groups of viruses, *Chlorella* viruses, *Marseilleviridae* viruses and *Phaeocystis globosa* viruses, encode R-M systems. In the list, these viruses are marked “Yes” in the column “Encodes R-M system”.

### Datasets of site-genome pairs

To study underrepresentation of R-M system recognition sites in corresponding genomes we used the following datasets. The first set, *the actual pairs dataset*, consists of all site-genome pairs where (1) the site and the prokaryotic genome are listed in the Additional file 1 and Additional file 2 correspondingly and (2) the genome encodes a REase that recognizes the site. In this paper, such sites are called ‘actual’ for the genome. The actual pairs dataset is available in Additional file 4. The list includes 3449 bacterial and 116 archaeal site-genome pairs.

An R-M system recognition site can be experimentally proven as well as predicted by REBASE. A predicted R-M system may be inactive or recognizing another site than predicted one [37]. We analyzed separately *the dataset of experimentally proven pairs*. The site-genome pair is considered to be experimentally proven if (1) the genome encodes an R-M system that is included in “Gold Standard Set” of REBASE [38] and recognizes this site, or (2) for the genome there are data on restriction site methylation obtained by Pacific Biosciences SMRT sequencing [10, 37, 39, 40] and the methylation level of the site is above 50 %. The dataset of experimentally proven pairs is a subset of the actual pairs dataset and is marked in Additional file 4.

To verify the results obtained for the actual pairs dataset we have also prepared two negative control datasets, namely *the viral control dataset* and *the prokaryotic control dataset.*

The viral control dataset consists of all recognition sites of R-M systems and all available sequences of eukaryotic viruses (Additional file 3) except the viruses that encode R-M systems (*Chloroviruses* and a couple of other viruses). We analyzed all possible pairs of viral genomes with restriction sites from Additional file 1. Eukaryotic virus genomes were used here as a negative control because eukaryotic viruses meet no R-M systems in their life. Nevertheless, usage of this set as a control dataset has some limitations. Viral genomes are significantly shorter than prokaryotic ones that may affect the statistical results. Even more important is a potential influence of certain other inevitable factors, differentiating viruses and prokaryotes.

The prokaryotic control dataset consists of all site-genome pairs where the site is listed in Additional file 1 and the genome is in Additional file 2. The actual pairs dataset is the subset of the prokaryotic control dataset. Moreover, some pairs from the prokaryotic control set may correspond to sites of yet unknown R-M systems. However the fraction of such pairs is negligibly small. We estimate it to be less than 1 % because about six hundred sites in each genome are included in the prokaryotic control dataset whilst there are only 2.6 R-M systems *per* genome on average according to data of Oliveira and co-workers [41], and 2.3 R-M systems according to our data.

The subset of site-genome pairs from the prokaryotic control dataset with genomes encoding no R-M systems have advantages of both control sets. Presumably, ancestors of these prokaryotes also met no R-M systems. At the same time, these genomes are prokaryotic genomes and thus have prokaryotic features that could affect sites number. However, this control set is much smaller than the prokaryotic control dataset (122 *vs.* 2114 genomes), that is why we mostly use the latter one in this work.

### Measurement of deviation of site occurrences number

Deviation of number of site occurrences was measured by the ratio *Kr* of the observed number of sites to the expected number. We used a formula suggested by Karlin *et al.* [42] for calculation of the expected number of sites. See (Additional file 5: Figure S1) for the formula for *Kr*. Also we calculated an analogous value *Mr* using another method, namely the maximum order Markov model [43]. Comparing distribution of two values for the control sets we found that the variance of *Kr* is less than the variance of *Mr* (see Additional file 5: Figure S2). Due to this observation and other arguments (see Discussion) we choose *Kr* as the main measure of deviation.

### Thresholds of under- and overrepresentation

We used the two-sample Kolmogorov–Smirnov test to compare distributions of *Kr* in two sets of site-genome pairs.

To obtain numbers of under- and overrepresented sites in different datasets we used as the first choice the *Kr* value cutoffs according to [42]: 0.78 for underrepresentation and 1.23 for overrepresentation. We roughly estimate percents of sites over- and underrepresented due to other causes besides R-M system influence as 1 % by comparison with the viral control dataset. Indeed, we found that 1.1 % pairs of the viral control dataset have *Kr* ≤ 0.78 and 1.0 % pairs have *Kr* ≥ 1.23. In the subset of site-genome pairs from the prokaryotic control dataset with genomes encoding no R-M systems there are 0.3 % of underrepresented sites with this threshold.

The thresholds of underrepresentation were adjusted for particular subsets of site-genome pairs (*e.g.* subsets with palindromic and asymmetric sites, see Results).

χ^2^ test or exact Fisher test were used for comparison of fractions of underrepresented sites in datasets.

In our work we considered only site-genome pairs for which the expected number of the site occurrence in the genome is more than 15. If the expected number of sites is small, then the ratio of the observed number of sites to the expected number of sites may be unreliable and thus should not be considered. To find the threshold for the expected number we performed the following procedure. For each range of expected number of sites we estimated the standard deviation of *Kr* by comparison of *Kr* value for each non-palindromic site with the same value for the complementary site. All recognition sites in all prokaryotic genomes with the expected number of sites within ranges 6.5 to 7.5, 7.5 to 8.5, *etc.* were considered. We found that starting from the range of 14.5 to 15.5 the standard deviation is less than 0.10 and decreases for larger expected numbers of sites. Thus if the expected number of sites is 15 or more, then the probability to observe *Kr <* 0.78 by chance is less than 0.012, and decreases rapidly.

### Identification of putative horizontally transferred restriction endonuclease genes

Putative horizontally transferred DNA fragments of prokaryotic chromosomes were predicted by Alien_hunter program with default parameters [44]. This program returns a score threshold and, for each putative horizontally acquired DNA, its coordinates and score. The score reflects the compositional deviation of putative horizontally transferred fragments from the genome background. A higher score corresponds to a more atypical fragment. We used scores over the threshold as a measure of reliability for detection of alien fragments.

The coordinates of REase genes were compared with coordinates of the obtained putative horizontally transferred fragments. If such fragment overlaps an REase gene, then we marked the gene as a putative recently acquired one and assigned the score of the fragment to the site-genome pair, where the site is the REase recognition site. If the genome encodes two or more REases recognizing the same site, then the minimum of their scores was assigned to the site-genome pair (assuming that fragments not predicted as putative alien ones have score 0).

## Results

### Avoidance of restriction sites in prokaryotic genomes depends on R-M system types

Taking into account different molecular organization of R-M systems and mechanisms of site recognition by REases, we classified the sites by the type of corresponding R-M systems (see Table 1) and analyzed site-genome pairs with the sites of each type in each dataset separately.

The sites of Type II REases were subdivided into Type IIC/G, Type IIM and the rest called ‘orthodox’ (see Materials and Methods). Type IIM sites were considered separately because Type IIM REases cleave only modified sites and are comparable to Type IV REases in that regard [5]. In contrast to orthodox R-M systems, Type IIC/G systems consist of a single protein that combines endonuclease, methyltransferase, and DNA recognition functions. Therefore Type IIC/G R-M systems are similar to Type I and Type III by structural and functional organization [45].

Percentages of site-genome pairs with the underrepresented sites for different R-M system types are shown in Table 2. Underrepresentation is stated if *Kr* is less or equal to 0.78. We observed site avoidance in the actual pairs dataset only for orthodox Type II sites and for GATC site of Type IIM methyl-directed REases, which is underrepresented in 38 of 42 cases. Nearly full avoidance of GATC site in this case is surprising, since methyl-directed R-M systems are presumably not deleterious for a bacterial genome if it is not methylated. We consider possible causes of GATC avoidance in Discussion.

**Table 2:** Percentage of underrepresented sites of different types in the different datasets

R-M system site type	Actual pairs dataset	Experimentally proven dataset	Prokaryotic control dataset	Viral control dataset
Type I	0.0 %	0.0 %	0.1 %	0.1 %
	0/100	0/14	238/357501	21/18859
Type III	0.0 %	0.0 %	0.3 %	0.2 %
	0/76	0/7	213/82065	57/31571
Type IIC/G	0.0 %	0.0 %	0.1 %	0.2 %
	0/107	0/47	171/218322	66/27699
Type II orthodox	47.9 %	45.3 %	3.9 %	1.7 %
	850/1774	58/128	21380/542911	2720/158921
Type IIM	70.4 %	14.3 %	0.6 %	0.3 %
	38/54^a^	1/7	125/21128	79/29070
Type IV	0.0 %	0.0 %	1.0 %	0.2 %
	0/13	0/3	64/6342	25/10116

^a^All 38 underrepresented sites are GATC

Eukaryotic viruses do not meet any R-M systems in their life usually and do not avoid any R-M system sites, see Table 2. However several eukaryotic viruses, i.e. *Chlorella* viruses, *Marseilleviridae* viruses and *Phaeocystis globosa* viruses encode orthodox Type II R-M systems. In these viral genomes the sites of the corresponding R-M systems are underrepresented in 47 % cases (8 cases of 17) (see Additional file 4).

In the prokaryotic control dataset, we analyzed the subset with genomes encoding no R-M systems at all. In this subset, sites of all R-M systems are underrepresented in only 0.3 % of all site-genome pairs and in 0.7 % site-genome pairs with orthodox sites.

**Fig. 1:**
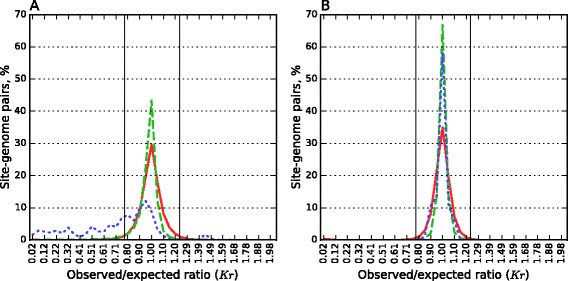
Histograms of *Kr* for various sets of site-genome pairs. There are 41 equal bins at the segment [0; 2), the percent of site-genome pairs with Kr falling into a bin is shown over its middle. **a** Blue dotted line shows the distribution for actual pairs of orthodox R-M-systems; green dashed line is for the subset of orthodox site-genomes pairs of the prokaryotic control set; red solid line is for the similar subset of the viral control set. **b** Blue dotted line shows the distribution for actual pairs of R-M-systems of types I, IIC/G, and III together; green dashed line is for the subset of non-orthodox site-genomes pairs of the prokaryotic control set; red solid line is for the similar subset of the viral control set. In both cases the cutoffs for under- and overrepresentation are shown by solid vertical lines

Figure 1 presents histograms of *Kr* for two subsets of the actual pairs dataset. The first subset consists of all site-genome pairs with orthodox Type II sites (Fig. 1a) and the second one consists of all pairs with sites of Types I, IIC/G and III grouped together (Fig. 1b). Histograms of *Kr* for prokaryotic and viral control datasets with the corresponding sites serve as a control.

The histogram of *Kr* for actual pairs with orthodox sites drastically differs from the histograms for both control sets (Fig. 1a); the latter ones basically coincide in the underrepresentation area. Note that in the figures (Fig. 1a and b) we restricted control sets to subsets of site-genome pairs with sites of considered groups (orthodox sites in Fig. 1a and non-orthodox sites, *i.e.*, of Types I, IIC/G, and III together in Fig. 1b). The difference between the *Kr* histograms for actual dataset of orthodox sites and the prokaryotic control dataset is especially demonstrative because both the list of sites and the list of genomes are the same. The only difference is that in the case of the prokaryotic control dataset all site-genome pairs are examined while in the case of the actual pairs dataset the genome is required to encode a REase recognizing this site.

Although the prokaryotic control dataset includes a certain fraction of actual pairs, this fraction is negligibly small (we estimate it to be less than 1 %) and thus the corresponding histogram well approximates *Kr* distribution for non-actual pairs in the case of prokaryotic genomes.

We found a site avoidance in 47.9 % actual pairs with orthodox sites (see Table 2). In the prokaryotic control dataset we found orthodox site avoidance only in 3.9 % cases. Many of these cases might be traces of lost R-M systems (see below). The *Kr* distribution for the subset of prokaryotic control dataset with genomes encoding no R-M systems is similar to that of the entire prokaryotic control dataset (Additional file 5: Figure S3). However, there are significantly less underrepresented site-genome pairs in the subset of site-genome pairs lacking R-M systems, namely 0.7 % *vs* 3.9 % in the entire prokaryotic control dataset for *Kr* < 0.78 threshold.

In contrast to orthodox Type II R-M system sites, *Kr* distribution for the joined subsets of the actual pairs of Types I, IIC/G, III practically does not differ from the distribution for the corresponding subset of the prokaryotic control dataset (Fig. 1b). A certain difference between the histograms in the area 0.8 < *Kr* < 0.95 is observed. We found that this difference is due to the contribution of only one site CAGAG of Type III REases from 20 *Salmonella enterica* strains. No significant difference was found between the histograms of the non-orthodox pairs (except CAGAG) in the actual pairs dataset and the prokaryotic control dataset in the area of underrepresentation (p > 0.05, Kolmogorov-Smirnov test, see also Additional file 5: Figure S4).

Our list of actual pairs includes a large amount of sites of predicted R-M systems. To estimate the effect of misannotation of sites, we analyzed site avoidance in the experimentally proven dataset. We found 45.3 % cases of site underrepresentation in the experimentally proven dataset with orthodox Type II R-M systems site. This value is close to 47.9 %, the overall percent of avoidance cases of the orthodox Type II R-M systems sites in the actual pairs dataset (see Table 2). Thus despite the fact that our set of annotated sites could contain some mispredictions, their influence on the results of recognition site avoidance seems to be negligible.

### Underrepresentation of palindromic and non-palindromic sites

**Fig. 2:**
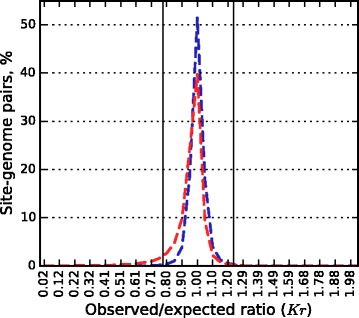
*Kr* distributions for palindromic (red dashed line) and non-palindromic (blue dashed line) sites of orthodox R-M systems for the prokaryotic control dataset

It was shown [30, 32, 42] that palindromes are the most underrepresented short words in a prokaryotic genome. This effect was mainly explained by R-M system presence because the recognition sites of the majority of orthodox REases are palindromes [29, 30, 32]. However even for control sets *Kr* distribution for palindromic and non-palindromic sites are different (Fig. 2).

**Fig. 3:**
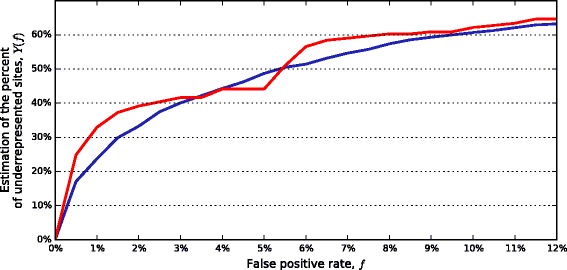
Graphs of site avoidance due to R-M system presence for palindromic (blue) and non-palindromic (red) sites. Fraction *f* of underrepresented sites in a subset of the prokaryotic control dataset (false positive rate) is on the X axis. The difference between percentage of underrepresented sites in the corresponding actual dataset and the false positive rate is on the Y axis. At each point of the graphs, *Kr* threshold for underrepresentation is fixed at the value giving *f*(%) of underrepresented sites in a control dataset

To compare the fractions of underrepresented palindromic and non-palindromic sites, this difference evidently should be taken into account. We consider *Kr* threshold *T*_*pal*_ for palindromes comparable with *Kr* threshold *T*_*n/pal*_ for non-palindromic sites if the fractions of underrepresented sites in the corresponding control datasets are equal; let us denote this common fraction by *f*. The fraction *f* can be considered as a fraction of false positives, i.e. sites underrepresented for reasons not related to R-M system presence in the genome. Thus, the difference *Y*_*pal*_*(f)* = *f*_*pal*_*– f* , where *f*_*pal*_ is the fraction of underrepresented sites in actual palindrome dataset, is an estimation of the fraction of sites underrepresented due to R-M system presence in a genome. The analogous value *Y*_*n/pal*_ is defined for the non-palindromic actual dataset. Figure 3 demonstrates that fractions of underrepresented sites in actual palindromic and non-palindromic datasets nearly coincide. Fluctuations of the graph *Y*_*n/pal*_*(f)* are likely caused by a rather small amount of actual site-genome pairs with non-palindromic sites (161 vs. 1613 palindromic ones).

**Fig. 4:**
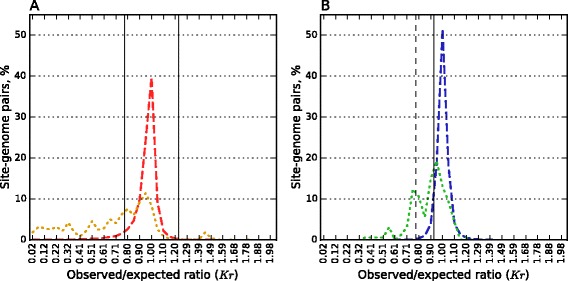
*Kr* distributions for palindromic (**a**) and non-palindromic (**b**) orthodox R-M system sites. Histograms for the prokaryotic control dataset are shown with dashed red (**a**) and blue (**b**) lines, for the actual pairs dataset with dotted orange (**a**) and green (**b**) lines. *Kr* thresholds are marked by vertical lines. The prokaryotic control datasets are the same as in Fig. 2

False positive rate *f* (%) = 5.5 % corresponds to the already used *Kr* threshold *T*_*pal*_ = 0.78 for palindromes and *T*_*n/pal*_ = 0.926 for non-palindromic sites (see Fig. 4a and b). Using these thresholds, we obtained 52.2 % underrepresented pairs in the dataset of actual pairs with non-palindromic sites and 50.5 % underrepresented actual pairs with palindromic sites.

We conclude that the fractions of orthodox recognitions sites avoided due to R-M systems presence in the genome are the same for palindromes and non-palindromes. At the same time palindromes generally are underrepresented in a greater degree than non-palindromes perhaps due to reasons that do not concern R-M systems.

### Other site peculiarities

We found no considerable difference in the avoidance for sites of different length or degeneracy.

There are a number of particular sites with outlying behavior. For example, there is only one site CTAG that is underrepresented in the majority of tested genomes (independently of corresponding R-M system presence in them). This phenomenon was described earlier [29–31], see also Discussion. At the same time there are 59 orthodox recognition sites underrepresented in the majority of those genomes where an R-M system with this site is annotated. Other outliers are CCGG and GATC (see below).

### Influence of lifespan of R-M systems on palindromic site underrepresentation

The reduction of the number of recognition sites in a prokaryotic genome needs many generations. Thus the influence of recently acquired R-M systems on the number of recognition sites can hardly be detected. This assumption was discussed in literature [32] and confirmed by Seshasayee *et al.* [46].

As it is shown above, the underrepresentation is observed almost only for orthodox Type II R-M systems, and we limited our analysis to such systems. To avoid the input of uneven distributions of *Kr* for palindromic and non-palindromic sites, we analyzed only palindromic sites because there are few non-palindromic sites for a detailed statistical analysis. To avoid any influence of site length, we considered only 4–6 bp palindromic sites, because they constitute the majority of all palindromic sites. Totally 1175 site-genome pairs were taken for the analysis.

It is impossible to directly measure the lifespan of an R-M system. Thus we used the following indirect approach. We divide (*via* different ways, see below) all actual site-genome pairs into two groups, where one group is presumably enriched with the sites of recently acquired systems and the other one is enriched with sites of long-lived in the genome R-M systems. The increase of fraction of underrepresented R-M system recognition sites in the ‘long-lived’ group can be considered as an evidence of the role of the lifespan of R-M systems.

We considered three ways to divide actual site-genome pairs into two groups. First, we separated R-M systems encoded on plasmids from chromosome-encoded ones. Significant fraction of plasmids are commonly horizontally transferred. Among them there are conjugative plasmids as well as non-conjugative ones [47]. Thus we may assume that plasmid-encoded R-M systems are enriched with recently acquired ones due to plasmids interventions into bacterial populations. The second way is to pick out chromosome-encoded R-M systems that are found in many related genomes; such systems are presumably long-lived. The third way is to take into account the difference of nucleotide composition in chromosome fragments encoding the R-M system from the average genome composition. Genes disposed in a fragment with a contrasting composition are likely to be recently horizontally transferred. The influence of the lifespan of an R-M system on the number of its recognition sites was observed for all above ways of division.

#### Plasmid-encoded R-M systems vs. chromosome-encoded ones

**Fig. 5:**
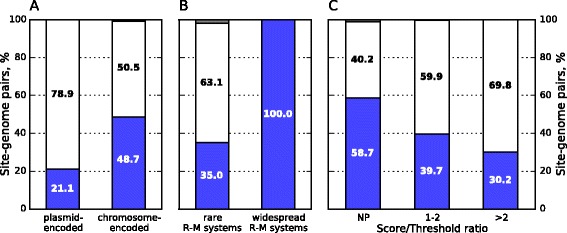
Sites representation of both recently acquired and old R-M systems in the actual site-genomes pairs dataset. Percentages of underrepresented, normally presented and overrepresented sites of: (**a**) plasmid-encoded and chromosome-encoded R-M systems, (**b**) rare and common R-M systems among chromosome-encoded ones, (**c**) R-M systems encoded in genomic fragments with various ratios of score to threshold calculated with Alien_hunter program, among chromosome-encoded that are neither rare nor common ones. NP is for R-M system genes encoded in the genomic loci that are not predicted by Alien_hunter as putative horizontally transferred ones. Blue color denotes underrepresented pairs (site, genome), grey color is for overrepresented pairs, pairs with *Kr* between 0.78 and 1.23 are in white. *Helicobacter pylori* genomes were excluded from the histograms (see the text)

We compared percentage of underrepresented sites in prokaryotic genomes for plasmid-encoded and chromosome-encoded R-M systems in the actual pairs dataset. The sites of plasmid-encoded R-M systems are avoided in corresponding bacterial genomes more rarely than of the chromosome-encoded ones: 21.1 % (of 38 cases) and 48.7 % (of 1137 cases) respectively (p < 0.001, χ^2^ test) (Fig. 5a). This result is in agreement with the assumption that sites of recently acquired R-M systems are less underrepresented (see Discussion).

#### Rare systems vs. widespread ones

Some R-M systems are encoded in the majority of strains of one species, other ones are encoded only in a few strains. We consider a chromosome-encoded R-M system as rare if R-M systems with the same specificity are annotated in less than 25 % species strains. R-M systems that are annotated in more than 75 % species strains are considered as widespread ones. Only species with five or more strains were analyzed. *Helicobacter pylori* strains were excluded from the analysis and analyzed separately (see below).

For 4–6 bp palindromes that are the sites of chromosome-encoded orthodox Type II systems in the actual pairs dataset we compared percentage of avoided sites in genomes for rare and widespread systems. Sites of rare R-M systems are avoided in 35.0 % (of 103) of cases, while the sites of widespread ones are avoided in 100 % (all 45) of cases (p < 0.001, χ^2^ test) (Fig. 5b).

*H. pylori* is an exception from the rule: in the actual pairs dataset the sites of rare R-M systems are avoided in 86.5 % of 37 cases, while the sites of widespread R-M systems are avoided in only 56.6 % of 235 cases. Moreover, 16.2 % sites of widespread R-M systems in the actual pairs dataset are even overrepresented. *H. pylori* strains are known outliers with respect to R-M systems [41] (also see below).

#### Recently horizontally acquired R-M systems

The comparison of rare and widespread R-M systems provides the evidence in favor of a high rate of site avoidance for ancient R-M systems, but the analysis concerned a small fraction of the available data, that is 148 actual site-genome pairs of 1137 ones with chromosome-encoded R-M systems. For the rest 989 chromosome-encoded actual site-genome pairs we used Alien_hunter program [44] for a prediction of REases encoded on chromosome regions of ‘alien’ origin. We found that recognition sites of Type II REases encoded in a genomic fragment with greater composition bias according Alien_hunter are underrepresented in the entire genome less often (see Fig. 5c). The relatively low sensitivity of the program (65 % according to the original publication) should be taken into account for a correct interpretation of this result.

These results confirm the assumption about the linkage between site underrepresentation and the lifespan of R-M system genes in a genome.

*Helicobacter pylori* strains deviate from the trend. Sites of R-M systems, encoded on putative recently acquired genome fragments (Alien_hunter score above the threshold) are underrepresented in 81.0 % cases (of 137). Sites of R-M systems encoded in the genome fragments that do not differ from the background are underrepresented in 47.6 % cases (of 290) and are overrepresented in 12.4 % cases (of 290).

### Traces of lost R-M systems

**Fig. 6:**
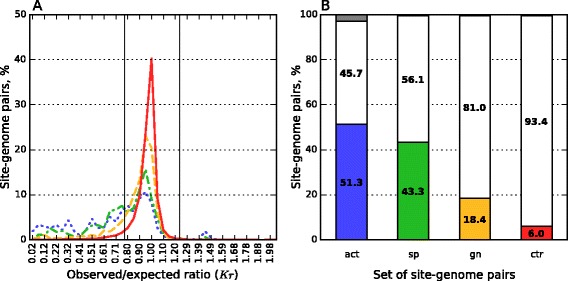
Representation of sites in genomes for cases the R-M system encoded only in related genomes. Only 4–6 bp palindromic recognition sites of orthodox Type II R-M systems are considered. **a** *Kr* distributions for site-genome pairs. Blue dotted line is for pairs of the actual pairs dataset, red solid line is for pairs of the prokaryotic control dataset. Green dash-dotted line is for sites of R-M systems that are not encoded in the current genome, but are present in some strain of the same species. Orange dashed line is for sites of R-M systems that are not encoded in any genome of current species, but are present in some species of the same genus. **b** Percentages of site representation. Colored part of stacked column denotes underrepresented sites, grey part of stacked column is for overrepresented ones, white part of stacked column corresponds to pairs with *Kr* for the site between 0.78 and 1.23. Colors correspond to (**a**). Legend: act — actual pairs, sp — actual in another strain of the same species, gn — actual in another species of the same genus, ctr — the prokaryotic control dataset

Prokaryotes often avoid the sites of R-M systems that are encoded in the genomes of their closest relatives, but not in their own genomes (Fig. 6). Sites of R-M systems that are not encoded in the genome but encoded in chromosomes of other strains of the same species are underrepresented in 43.3 % (of 1930) of cases, and those encoded in chromosomes of other species of the same genus in 18.4 % (of 5162) of cases, as compared to 6.0 % (of 339646) of underrepresented cases for all 4–6 length palindromes in all prokaryotic genomes from the prokaryotic control dataset. In other words, closely related genomes share similar underrepresented sites pattern regardless of encoded R-M systems. These results confirm earlier discussed assumptions [30, 32] that the site avoidance could be a vestige of lost R-M systems.

We similarly analysed 38 actual site-genome pairs of plasmid-encoded R-M systems. These R-M systems belong to 35 species of 30 genera. Among them 15 R-M systems are encoded in species that have one strain with the plasmid-encoded R-M system and other strains without this R-M system. For 13 of them corresponding sites are underrepresented in only 9.5 % (4 of 42 cases) strains without the R-M system. Two remaining plasmid-encoded R-M systems, EcoCE10ORF1P and EcoO26ORF5P, belong to *E. coli* strains. Sites of EcoCE10ORF1P are not avoided in any strain not encoding this R-M system. Sites of EcoO26ORF5P are avoided in all 56 *E. coli* strains while the plasmid encoding it is known only for one strain.

It is likely that R-M systems encoded on mobile plasmids typically have short lifespans and do not leave noticeable traces in visited genomes. At the same time, certain plasmids are long-living and not mobile [47]; the plasmid encoding EcoO26ORF5P could be of such kind. Thus these results are also in agreement with the lifespan influence on the site avoidance.

It should also be noted that according to our data there are much more underrepresented sites than annotated R-M systems per genome in the prokaryotic control dataset. This can be partly explained by unannotated R-M systems and partly by traces of lost R-M systems.

We estimated roughly the fraction of avoided sites which are the traces of lost orthodox R-M systems. In prokaryotic genomes that do not encode any R-M systems only 0.7 % of all 29318 orthodox site-genome pairs are underrepresented. This percentage allows us to estimate the site avoidance which results from other reasons than R-M systems. If so, the majority of underrepresented sites in the prokaryotic control dataset are traces of lost R-M systems. Thus the avoidance of the sites of lost R-M systems is widely spread in prokaryotic genomes.

### Site overrepresentation

We found 47 cases of overrepresentation of orthodox Type II R-M systems sites in the actual pairs dataset (the peak in Fig. 1a for *Kr* ~ 1.4–1.5). In 41 of these cases the site is CCGG and the genome is one of genomes of *Helicobacter* genus (38 strains of *H. pylori*, one strain of *H. acinonychis*, and two strains of *H. cetorum*).

Activity of R-M system genes recognizing CCGG site was proven to a restricted number of *H. pylori* strains. REase activity was shown in *H. pylori* strain ATCC 49503 [48]; the activity of MTase was shown in *H. pylori* strain J99 [49]. The corresponding genes are inactive in *H. pylori* strain 26695 [50]. Although other *H. pylori* R-M genes recognizing CCGG in our dataset are marked ‘predicted’ in REBASE, the great number of them suggests that overrepresentation of CCGG sites is unlikely to be associated with inactive R-M systems.

Overrepresentation of CCGG sites might be explained by a positive effect of their methylation with MTases that overrides negative effect of probable DNA cleavage with REases. One of possibilities for the positive effect of the methylation is an influence on the gene expression. A correlation between the methylation and the regulation of gene expression was studied by Vitoriano et al. [51] for 30 *H. pylori* strains. The site CCGG was not found among the sites whose methylation status affects gene expression [51].

Thus we have no reasonable biological explanation for CCGG overrepresentation in the *H. pylori* genomes.

## Discussion

The avoidance of palindromic restriction sites in prokaryotic genomes was demonstrated earlier for several dozens of R-M systems [29, 30, 32]. Here we present, for the first time, the results of a full-scale analysis of the phenomenon. Importantly, the current amount of R-M systems with annotated restriction sites exceeds 3500 in REBASE. Thus we were able to study the avoidance of restriction sites directly and systematically, no matter how long are they, are they degenerate or not, palindromes or not. This is in contrast to the previous works, where restriction sites were selected from the lists of avoided non-degenerate palindromes [29, 30, 32, 33] or words of certain length [31, 52].

Methods of site avoidance detection and estimation can significantly influence the results of the analysis [53]. All of them are based on the comparison of the number of occurrences of an examined word in a genome with the statistically expected number of its occurrences. Various methods for the estimation of an expected number of word occurrences were used in literature. Simpler methods consider base composition only [46], more sophisticated ones take into account a number of occurrences in a genome of certain subwords of the word [42, 43, 54]. Elhai [53] compared these methods applied to *E. coli* genome and concluded that the results of different methods can greatly vary from each other, and Karlin’s method [42] is more reliable.

We have additional arguments to use Karlin’s method in our work. This method takes into account frequencies of all subwords including degenerate ones. This approach seems to be appropriate for studying R-M system recognition sites because half of them are degenerate. Higher precision of Karlin’s method in comparison with the commonly used method based on maximal order Markov model [43] is indirectly confirmed by less variance of the expected to observed number ratio for the former (Additional file 5: Figure S2).

As a measure of site underrepresentation (or overrepresentation) we used the ratio *Kr* of the observed number of sites to the expected one according to Karlin’s model. We presume that the ratio is a better measure of selection pressure than used in a number of works [30, 32] p-value because the latter significantly depends on genome size. At last, it is not possible to compute p-value for the deviation of the observed number of sites from the expected one according to Karlin’s method because, as far as we know, no mathematical results on this subject were published.

Like in previous works, we consider R-M site avoidance (*i.e.*, a selection in favour of decreasing the number of sites) in the case of site underrepresentation, *i.e.*, if the *Kr* value is less than a threshold. Certainly, the threshold choice influences the fraction of underrepresented sites in the cohort of site-genome pairs under our study. The usage of control datasets allowed us to substantiate the choice and also to estimate the fraction of false positives among the cohort of underrepresented sites.

Our approach can not detect site avoidance for the R-M systems recently transferred into host genome or changed their specificity, because the *Kr* value clearly depends on selection pressure as well as on the lifespan of an R-M system.

We found the avoidance for the sites of orthodox Type II R-M systems approximately in half of the cases. No avoidance was observed for non-orthodox sites, *i.e.*, the sites of Type I, Type III and Type IIC/G and methyl-directed Type IIM and Type IV, except of the GATC site of Type IIM R-M systems and CAGAG of Type III R-M systems.

Site avoidance in prokaryotic genomes for orthodox R-M systems agrees with our expectations and results of previous works [29, 30, 32]. This effect was attributed to cleavage of prokaryotic DNA by restriction endonucleases due to incomplete methylation of DNA sites [30–32]. On the other hand, genome methylation by methyltransferases from R-M systems can affect gene expression [9, 37]. Thus it is likely that selection is addressed primarily against recognition sites, methylation of which is unfavorable for bacteria.

The absence of site underrepresentation is surprising for non-orthodox non-methyl-directed R-M systems. Indeed, all types of R-M systems (except methyl-directed ones) are well known to be toxic for unmethylated foreign DNA [28]. So a similar self-toxicity could be expected for all types of R-M systems.

We hypothesize that both less self-toxicity and shorter lifespan of non-orthodox R-M systems contribute to the failure to detect site underrepresentation. First, restriction endonucleases from non-orthodox R-M systems become active only in complex with cognate MTases. Thus in the case of MTase failure (such as a loss of its ability to recognize sites or to form an active endonuclease complex) the only consequence is the loss of defence function. In contrast, REases and MTases of orthodox Type II R-M systems act independently from each other. Thus MTase failure results in cleavage of the prokaryotic DNA by the corresponding REase. It makes the orthodox Type II R-M systems more toxic for a prokaryotic host. We conclude that non-orthodox R-M systems could be considered less self-toxic.

Second, non-orthodox R-M systems utilize the same DNA recognition domain for both endonuclease and DNA methyltransferase complexes. Therefore, the change of their recognition sequences is due to mutations in the single DNA recognition domain. Consequently, an active R-M system is retained [55, 56]. Mechanisms for frequent and even programmed (leading to phase variation) specificity changes were shown for Type I and III R-M systems [7, 11]. Fast recognition site evolution of Type IIC/G and Type IV genes was also shown [23, 41]. In contrast, orthodox Type II R-M systems require two simultaneous concordant mutations in two different genes (REase and MTase) for coordinated change of their specificity. Thus, specificity changes seem to be much rarer for orthodox R-M systems than for all others. As a result, selection has more time to eliminate the sites of orthodox R-M systems than of non-orthodox ones.

In the attempt to explain the drastic difference of site underrepresentation among orthodox R-M systems we examined the role of site features, their palindromicity, length and degeneracy, because the sites of R-M systems of different types can be distinguished by these parameters.

In our dataset 90 % sites of orthodox R-M systems are palindromes. Considering palindromic and non-palindromic sites of orthodox R-M systems separately, we found that among 161 non-palindromic sites of orthodox R-M systems the percentage of the avoided sites is almost the same as among palindromic ones. Note that *Kr* thresholds were chosen separately for palindromic and non-palindromic sites because palindromic sequences are more underrepresented than non-palindromic ones, regardless of the existence of active R-M systems in genomes (see Fig. 4).

We found that orthodox R-M system sites of a half of actual site-genome pairs are underrepresented in the corresponding genomes. Hence sites of the other half of pairs are not underrepresented.

Most likely the majority of non-underrepresented sites of actual site-genome pairs are the sites of recently acquired R-M systems. A lot of time is required for the site underrepresentation to become detectable. A smaller fraction of underrepresented sites of R-M systems in horizontally transferred regions (as compared to the ‘old’ ones) was shown in the work [46].

In this work we demonstrated that the frequency of underrepresentation for orthodox R-M system sites in the datasets presumably enriched with recently acquired R-M systems (Fig. 5) is two-three times lower. Especially demonstrative is the 100 % site avoidance detected in the group of 4–6 bp length palindromic sites of the orthodox R-M systems that are chromosome-encoded in the majority of strains of a species. It is likely that in these cases the R-M systems were at least in the common ancestor of strains and thus lifespan of these R-M systems is rather long. This observation confirms that a significant part of non-underrepresented sites of the orthodox R-M systems could be the sites of recently acquired R-M systems. There should be a certain caution in the interpretation of this data as the number of cases in the group with 100 % site avoidance is only 45.

Some other reasons for non-underrepresentation of orthodox R-M system sites should also be taken into account. It is well known that REases differ in their efficiency, therefore selection pressure against the sites may vary significantly. For example, BbrIII is about 200 times more effective than BbrI [57]. Besides, self-toxicity may depend on specific properties of sites distribution in a genome. In several cases, REases efficiency can depend on the number of restriction-modification sites, for example Ecl18kI, EcoRII, *etc.* are more active when DNA possesses several restriction sites [58, 59]. For several REases such efficiency depends on the site context [60–63] or peculiarities of sites distribution between DNA chains [28].

We also found a large amount of cases when a site is avoided in a particular genome while an R-M system with this site is not encoded in this genome. In many cases such system is encoded in a closely related genome, and we may assume that the R-M system was recently lost by the organism. These data confirm that some underrepresented sites are the vestiges of lost R-M systems (this statement was supposed earlier, see, for example, [32]). We speculate that the majority of underrepresented sites could be traces of lost orthodox R-M systems.

**Fig. 7:**
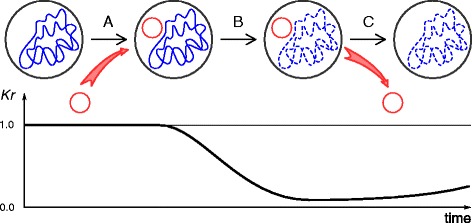
Model of R-M system influence on the underrepresentation of its site. **a** denotes the moment of acquiring a new R-M system. **b** is the persistence period of the R-M system in the genome, which leads to the underrepresentation of its site. **c** is the period after the loss of the R-M system. The red circle is for R-M system genes, the blue line is for the genomic DNA, the dashed blue line is for the genomic DNA that lost some recognition sites of the R-M system

Based on our data we propose the following model of R-M system effect on the number of its site in a bacterial or archaeal genome (Fig. 7).

Acquiring a new R-M system a bacterium gets the effective defense tool against bacteriophages. Due to this advantage the R-M system spreads fast in the bacterial population. At the same time the R-M system self-toxicity results in the decrease of the restriction site number in the host genome. The same process of restriction sites elimination may occur in phage genomes, probably in parallel with obtaining or adjusting other antirestriction mechanisms.

Long term bacterium – phage – R-M system coevolution and arms race result in their mutual adaptation. As soon as the phage has adapted to the R-M system, this system does not defend the bacterium anymore and becomes useless [28]. The lifetime of useless genes in bacterial genomes is relatively short [64, 65]. Thus the genes of the R-M system that has become ineffective would soon be corrupted and lost. However, the R-M system recognition site avoidance would remain in the bacterial genome for a long time.

The decrease of the number of R-M system recognition site occurrences in a genome requires a huge amount of generations. A restoration of site occurrence number after a loss of an R-M system is an even longer process. Its duration is likely to be similar to the time of the base composition restitution of horizontally transferred genes. Lawrence and Ochman estimated this time to be on the order of magnitude of hundred million years [66].

It is of special interest to analyze the outliers with specific features among restriction sites. The GATC site is the only underrepresented site among the sites of methyl-directed R-M systems. GATC is known to be the recognition site of a number of IIM REases (so-called DpnI-like) as well as of many orthodox Type II R-M systems. This site is underrepresented in most genomes (38 of 42) where corresponding methyl-directed R-M systems are encoded. Surprisingly, GATC avoidance in a genome carrying Type IIM R-M system is often accomplished with the presence of GATC-recognizing orthodox Type II R-M system genes in other strains of the same species (S*treptococcus pneumoniae, Neisseria meningitidis, Eubacterium rectale)* or genus (*Moraxella catarrhalis, Sulfurospirillum deleyianum)*.

For example, in *S. pneumoniae* there are strains encoding DpnII-like R-M systems, which cleave unmethylated GATC and methylate their own DNA, as well as strains encoding Type IIM DpnI-like R-M systems, which cleave methylated GATC [67]. Such complementary DpnI/DpnII-like systems can defend mixed bacterial population from phage attacks more effectively [68, 69].

However, such systems might prevent DNA exchange between strains with the different methylation status of GATC. To facilitate horizontal gene transfer, genomes of *S. pneumoniae* with Type II DpnII-like R-M system encode an additional methyltransferase, which methylates GATC in the single-strand DNA and thus prevents degradation of the acquired unmethylated DNA [70, 71].

We suppose that the GATC avoidance also facilitates DNA exchange between the strains with different methylation status of GATC.

The avoidance of GATC site can be also associated with activity of the methyl-directed mismatch repair system. Unmethylated GATC is target for the MutH nicking enzyme [72]. The toxicity of MutH for enterophage DNA with unmethylated GATC was shown [73]. All strains with the GATC-recognizing Type IIM R-M systems are dam**-** and therefore mismatch repair system might be the reason of GATC avoidance.

The other outlier is CTAG site which is the most avoided 4 bp palindrome in the bacterial genomes [29]. In our data it is underrepresented in 55.4 % of all site-genome pairs of prokaryotic control dataset while only 0.9 % of bacteria are known to have the R-M system with such specificity. The CTAG avoidance was explained by its structural role in DNA [29] (note, that the central dinucleotide TA is underrepresented in almost all genomes of all branches of life [31, 74]). Also it could be explained by the VSP repair system that is likely to turn CTAG into CCAG after A to G transition [29, 75, 76]. Furthermore CTAG is often the target site for insertion sequences [77] and can be under negative selection to prevent their expansion in a genome. Thus underrepresentation of CTAG can hardly be attributed exclusively to influence of orthodox Type II R-M systems.

## Conclusions

The phenomenon of restriction sites avoidance was discovered earlier for palindromic sites of Type II R-M systems. We showed that, with respect to the site avoidance, the R-M systems have to be divided into ‘orthodox’, which are Type II R-M systems except IIG and IIM subtypes, and the remaining, ‘non-orthodox’ ones. The sites of orthodox R-M systems are underrepresented in about 50 % of bacterial genomes encoding corresponding R-M system genes while site underrepresentation practically was not detected for non-orthodox R-M system sites. Many of non-underrepresented orthodox R-M system sites are likely to be the sites of recently acquired R-M systems.

As to non-orthodox R-M systems the absence of recognition site underrepresentation may be explained by a significantly shorter lifespan in bacteria due to efficient mechanisms of specificity changes, or less self-toxicity of such R-M systems. Indeed, methyltransferase corruption may prevent DNA cleavage because endonuclease complex in these group of R-M systems includes methyltransferase subunit.

Our data confirms also that underrepresented sites that are not sites of any orthodox R-M system encoded in a prokaryotic genome often are restriction sites of lost R-M systems. The underrepresentation of sites may be considered as a specific kind of traces of orthodox R-M system activity in a bacterial cell.

Systematic study of site avoidance shed light on R-M system evolution, mobility and interaction with host genomes.

## Additional files

Additional file 1:
**List of sites of all R-M systems available in REBASE.** Tab-delimited table of recognition sites of R-M systems that were analyzed in the work. (TSV 162 kb) Additional file 2:
**List of all analyzed prokaryotic genomic sequences.** Tab-delimited table of chromosomes and plasmids of complete prokaryotic genomes that were analyzed in the work. (TSV 445 kb) Additional file 3:
**List of all analyzed genomic sequences of eukaryotic viruses.** Tab-delimited table of viral genome sequence IDs. (TSV 191 kb) Additional file 4:
**The actual pairs dataset.** Tab-delimited table of site-genome pairs where site is the recognition site of one of R-M systems encoded in the genome. (TSV 435 kb) Additional file 5: Figure S1. Formula for *Kr* calculation by Karlin’s method. **Figure S2.** Distribution of *Kr* and *Mr* in the prokaryotic control dataset. **Figure S3.** Distribution of *Kr* for the orthodox sites from the prokaryotic control dataset (red line) and the subset of prokaryotic control dataset with genomes encoding no R-M systems (blue line). **Figure S4.** Distribution of *Kr* for the non-orthodox sites except CAGAG from the actual pairs dataset (blue line) and the prokaryotic control dataset (green line). (PDF 165 kb)

## Abbreviations

R-M system: restriction-modification system
MTase: DNA methyltransferase
REase: restriction endonuclease
