# Supplementary material for: Lifespan of restriction-modification systems critically affects avoidance of their recognition sites in host genomes
Source: BMC Genomics. 2015 Dec 21;16:1084. doi: 10.1186/s12864-015-2288-4 (PMC4687349; doi:10.1186/s12864-015-2288-4)
Supplement: Additional file 5: Figure S1. — Formula for Kr calculation by Karlin’s method. Figure S2. Distribution of Kr and Mr in the prokaryotic control dataset. Figure S3. Distribution of Kr for the orthodox sites from the prokaryotic control dataset (red line) and the subset of prokaryotic control dataset with genomes encoding no R-M systems (blue line). Figure S4. Distribution of Kr for the non-orthodox sites except CAGAG from the actual pairs dataset (blue line) and the prokaryotic control dataset (green line). (PDF 165 kb) [file 12864_2015_2288_MOESM5_ESM.pdf]

$$Kr(w) = \prod_{s \in S} F(s)^{(-1)^{(L(w)-L(s))}}$$

Figure S1. Formula for *Kr* calculation by Karlin's method. Notation: S is the set of all subwords of the word w, including w itself, F(s) is the observed frequency of the word s, L(s) is the length of s. Example formula for the word ATAT (N is any letter):

$$Kr(\texttt{ATAT}) = \frac{F(\texttt{ATAT}) \cdot F(\texttt{NNAT}) \cdot F(\texttt{NTNT}) \cdot F(\texttt{NTAN}) \cdot F(\texttt{ANNT}) \cdot F(\texttt{ANAN}) \cdot F(\texttt{ATNN})}{F(\texttt{ATAN}) \cdot F(\texttt{ATNT}) \cdot F(\texttt{ANAT}) \cdot F(\texttt{NTAT}) \cdot F(\texttt{ANNN}) \cdot F(\texttt{NTNN}) \cdot F(\texttt{NNAN}) \cdot F(\texttt{NNNT})} =$$
$$= \frac{F(\texttt{ATAT}) \cdot F(\texttt{AT})^2 \cdot F(\texttt{TNT}) \cdot F(\texttt{TA}) \cdot F(\texttt{ANNT}) \cdot F(\texttt{ANA})}{F(\texttt{ATA}) \cdot F(\texttt{ATNT}) \cdot F(\texttt{ANAT}) \cdot F(\texttt{TAT}) \cdot F(\texttt{A})^2 \cdot F(\texttt{T})^2}$$

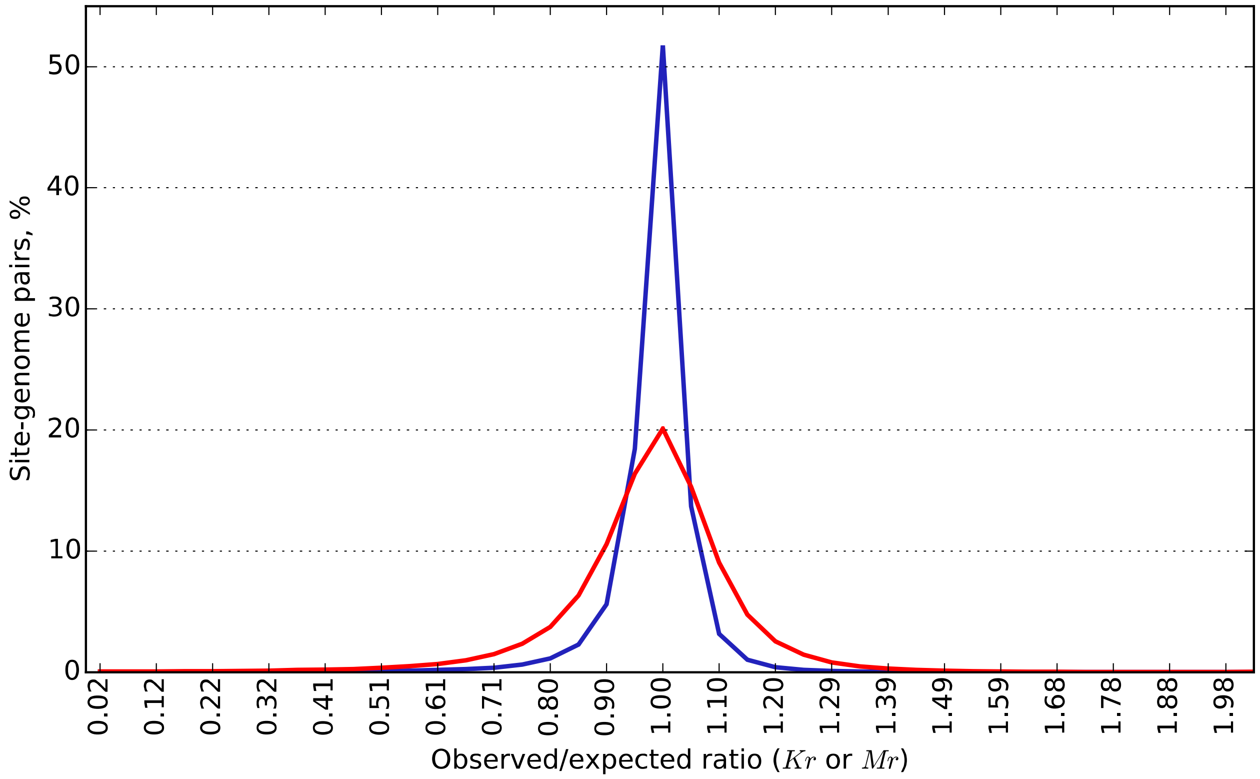

Figure S2. Distribution of *Kr* (blue line), and *Mr* (red line) for potential sites in bacterial genomes.

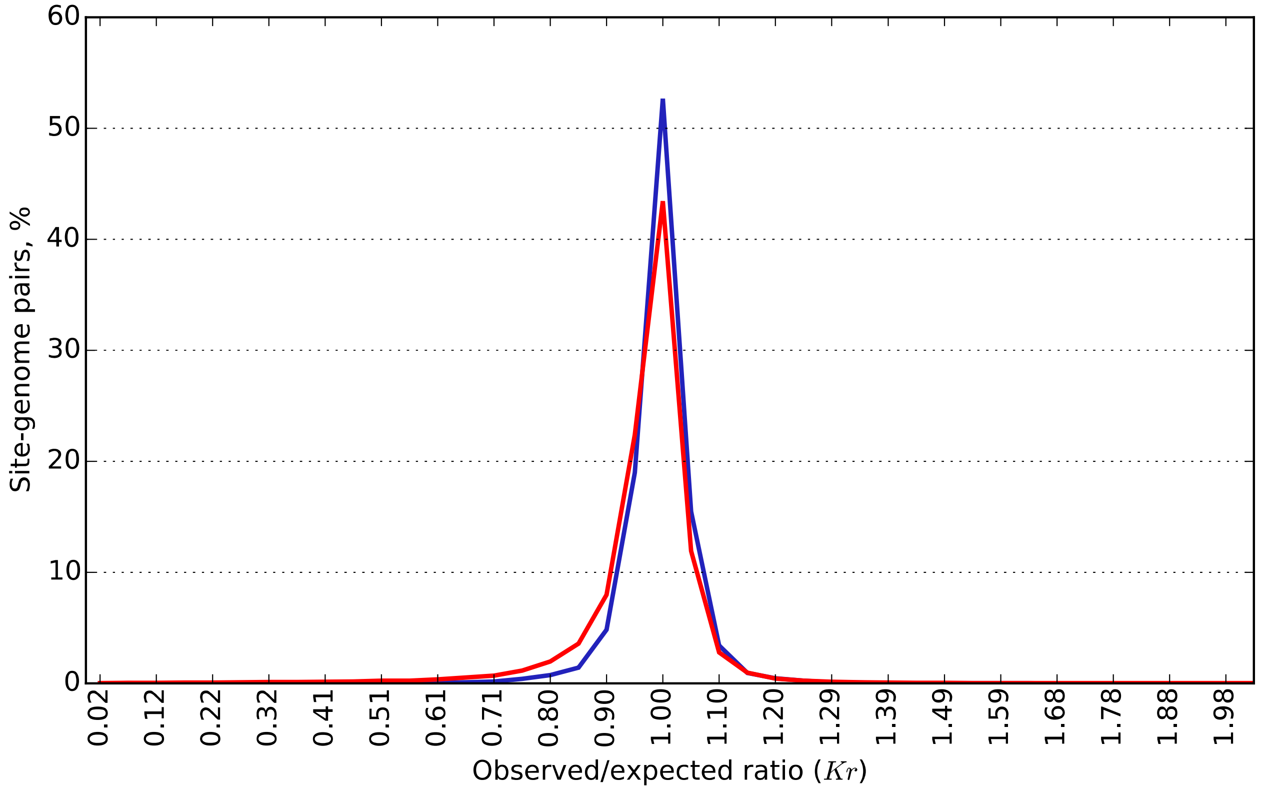

Figure S3. Distribution of *Kr* for the orthodox sites from the prokaryotic control dataset (red line) and the subset of prokaryotic control dataset with genomes encoding no R-M systems (blue line).

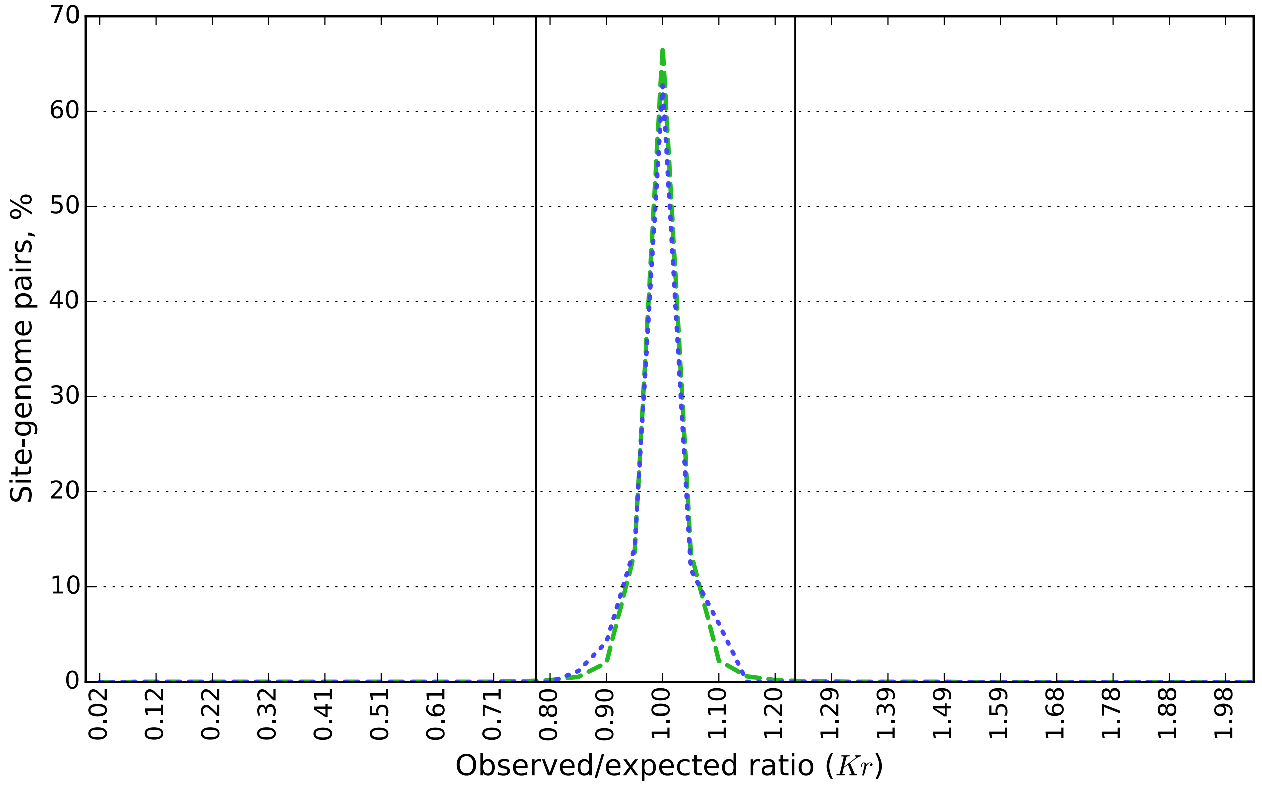

Figure S4. Distribution of *Kr* for actual (blue line) and potential (green line) non-orthodox sites except CAGAG.
